# OP13 Prioritizing Real-World Data In Next-Generation Health Technology Assessment: Insights From Oncotype DX Breast Recurrence Score® And Chemotherapy Recommendations For Breast Cancer In Brazil

**DOI:** 10.1017/S026646232510072X

**Published:** 2025-12-29

**Authors:** Carlos Alberto Magliano, Leandro Jonata de Carvalho Oliveira, Tomás Reinert, Carlos Henrique dos Anjos, Daniela Dornelles Rosa, Julio Antonio Pereira de Araújo, Andrea Kazumi Shimada, Daniele Assad, Marcelle Goldner Cesca, Max Mano, Gustavo Povoa

## Abstract

**Introduction:**

Mounting evidence shows that gene expression signatures in early breast cancer enable more precise chemotherapy recommendations, targeting those most likely to benefit and safely avoiding it for others. International guidelines highlight their role in optimizing adjuvant chemotherapy decisions. This study examined chemotherapy recommendation patterns in Brazil, providing decision-makers with locally specific data to support evidence-based and patient-centered care.

**Methods:**

We conducted an online survey with 10 independent clinical oncology experts, blinded to each other’s responses, to evaluate average chemotherapy recommendations considering age, menopausal status, and clinical risk assessment using the MINDACT study criteria with and without the Oncotype DX Breast Recurrence Score test. For node-negative patients, we analyzed recommendations by clinical risk level (high versus low), age category (over or under 50 years), and recurrence score (RS) ranges (<11; 11 to 25; >25). For node-positive patients, menopausal status and RS ranges (<14; 14 to 25; >25) were evaluated. The results were summarized as the arithmetic means of observed responses.

**Results:**

Chemotherapy recommendations were as follows.

- For node-negative patients under 50 years by clinical risk (low/high risk): Without Oncotype DX: 36 percent/89 percent; with Oncotype DX and RS less than 11: zero percent/eight percent; RS 11 to 25: 41 percent/70 percent; RS greater than 25: 88 percent/98 percent.
- For patients older than 50 years without Oncotype DX (low/high risk): 17 percent/77 percent; with Oncotype DX and RS less than 11: zero percent/six percent; RS 11 to 25: one percent/30 percent; RS greater than 25: 88 percent/98 percent.
- For node-positive patients: Premenopausal women had high chemotherapy rates (82 to 100%) regardless of RS, while in postmenopausal women rates ranged from three percent (RS<11) to 94 percent (RS>25), compared with 81 percent without testing.

**Conclusions:**

Oncotype DX Breast Recurrence Score reduced chemotherapy use in node-negative and postmenopausal node-positive patients, improving treatment targeting. This study revealed differences in chemotherapy recommendations between local Brazilian data and prior Oncotype DX models. Next-generation HTA should prioritize local realities to avoid misrepresentations that could misguide policymakers and to support sustainable and personalized healthcare frameworks in Brazil.

